# A Clinically Evaluated Interferometric Continuous-Wave Radar System for the Contactless Measurement of Human Vital Parameters

**DOI:** 10.3390/s19112492

**Published:** 2019-05-31

**Authors:** Fabian Michler, Kilin Shi, Sven Schellenberger, Tobias Steigleder, Anke Malessa, Laura Hameyer, Nina Neumann, Fabian Lurz, Christoph Ostgathe, Robert Weigel, Alexander Koelpin

**Affiliations:** 1 Institute for Electronics Engineering, Faculty of Engineering, Friedrich-Alexander University Erlangen-Nürnberg, Cauerstraße 9, 91058 Erlangen, Germany; kilin.shi@fau.de (K.S.); fabian.lurz@fau.de (F.L.); robert.weigel@fau.de (R.W.); 2 Chair for Electronics and Sensors Systems, Brandenburg University of Technology, 03046 Cottbus, Germany; sven.schellenberger@b-tu.de (S.S.); alexander.koelpin@b-tu.de (A.K.); 3 Department of Palliative Medicine, Medical Faculty, Friedrich-Alexander University Erlangen-Nürnberg, 91054 Erlangen, Germany; Tobias.Steigleder@uk-erlangen.de (T.S.); Anke.Malessa@uk-erlangen.de (A.M.); laura.hameyer@fau.de (L.H.); nina.neumann@fau.de (N.N.); Christoph.Ostgathe@uk-erlangen.de (C.O.)

**Keywords:** continuous wave radar, remote sensing, six-port interferometry, vital parameter measurement

## Abstract

Vital parameters are key indicators for the assessment of health. Conventional methods rely on direct contact with the patients’ skin and can hence cause discomfort and reduce autonomy. This article presents a bistatic 24 GHz radar system based on an interferometric six-port architecture and features a precision of 1 µm in distance measurements. Placed at a distance of 40 cm in front of the human chest, it detects vibrations containing respiratory movements, pulse waves and heart sounds. For the extraction of the respiration rate, time-domain approaches like autocorrelation, peaksearch and zero crossing rate are compared to the Fourier transform, while template matching and a hidden semi-Markov model are utilized for the detection of the heart rate from sphygmograms and heart sounds. A medical study with 30 healthy volunteers was conducted to collect 5.5 h of data, where impedance cardiogram and electrocardiogram were used as gold standard for synchronously recording respiration and heart rate, respectively. A low root mean square error for the breathing rate (0.828 BrPM) and a high overall F1 score for heartbeat detection (93.14%) could be achieved using the proposed radar system and signal processing.

## 1. Introduction

Automatic recording of vital parameters (VP) as a surrogate for health is part of clinical standard procedures, e.g., heartbeat by electrocardiography (ECG), pulse rate and oxygenation of the blood by pulseoxymetry and blood pressure by intermittent sphygmomanometry. These procedures serve many purposes, e.g., diagnostics, risk stratification and monitoring, and finally lead to better clinical outcomes in various settings [1,2,3]. In case of primary admission, gathering data on VP may help in identifying patients at risk and in arriving at the diagnosis and its differential diagnoses from a very early stage, e.g., through determination of the affected organs [4]. Beyond momentary assessment, repeated recording of VP may serve to adapt treatment of the patient. Detecting changes of VP may help in risk stratification and consecutively lead to tailored degrees of medical surveillance according to individual needs [5] and timely treatment in case of deterioration [6,7,8]. Complications and sudden deteriorations are most often unexpected in hospitals. In retrospective analyses of in-hospital deaths, less than 30% were anticipated and countered by intensive care treatment and over 70% occurred unexpectedly [9]. Continuous monitoring may help to avoid unexpected deaths and to achieve a better outcome by timely intervention. Long-term automated recording of VP yields additional great health benefits [10,11]. Various changes in VP that hint to potentially severe diseases occur only intermittently and thereby escape momentary diagnostics [12,13]. Automatically recording and evaluating VP based on predetermined limits allows continuous monitoring of health with affordable resources and early diagnosis of intermittent diseases [14].

Up to now, established VP recording is based on touch-dependent sensor technology. This kind of sensor technology has its disadvantages and limitations. Mobility and autonomy are reduced significantly for those in need of care. Additionally, skin irritations may occur when using adhesive electrodes and the constant unfamiliar sensation caused by sensors and cables may lead to discomfort and manipulation of the sensors, false alarms and an increased workload for clinical staff. Compliance of patients to use touch-dependent monitoring is further limited as the obvious presence of a medical device means emphasizing their illness and has an impact on social participation [15,16]. In cases of patients suffering from dementia or delirium, fixation measures may have to be ordered to ensure monitoring although external fixation itself promotes confusion and reduces quality of life [17].

Monitoring methods in hospitals and at home have to be chosen based on their benefits and disadvantages. For some patients the setting of an intensive or intermediate care unit with various degrees of invasive monitoring is favorable in terms of their personal outcome and in view of societal challenges regarding limited resources [18]. A large collective of patients would profit from an adaptable and non-invasive form of monitoring [19], especially if this kind of monitoring could bridge the gap between hospital and home care. To address this challenge adequately, the monitoring device should be touch-less, allow easy set-up and intuitive operation and has to capture basic VP, which are valid surrogate parameters for overall health, e.g., heartbeat and breathing.

To address the need of contactless VP monitoring, engineers have developed and investigated various novel concepts in the past years [20]. Using a high-resolution thermal camera, for instance, the heart rate of a person under test (PUT) can be extracted by sensing the temperature modulation of the skin in vicinity to blood vessels [21]. However, direct line-of-sight (LOS) between camera and PUT is required. While the results are very promising regarding respiration rate, the accuracy of the heart rate is limited to a root mean square error (RMSE) of several beats per minute [22]. This is sufficient for a coarse estimate, but does not satisfy medical standards [23]. Similar results can be obtained by monitoring the body movements with commercial cameras using the spectrum of visible and near infrared light [24,25]. Since LOS between sensor and PUT cannot always be guaranteed in clinical environments due to clothes and bedding, capacitive concepts were developed and realized as electrode arrays included in the mattress [26,27]. By this, accurate results can be obtained at the expense of a reduced comfort for the PUT due to the modified bedding.

Without requiring any electrodes near the PUT, heart rate and respiration rate can also be measured using microwave radar systems, which are able to accurately detect relative or absolute distances. Displacements of the body surface caused by these vital signs can hence be measured and used for an estimation of the VP. Since clothes and bedding can be penetrated, a direct LOS is not required. Prototypes have been built at different frequencies and modulations, most of them between 2.4GHz and 60GHz. To process the data, frequency domain methods like the evaluation of the fast Fourier transform or correlations of the filtered pulse wave are commonly used [28]. However, recent advances in research have shown that heart sounds can be recorded with highly precise six-port based radar systems by filtering the distance signal in a band between 16 Hz and 80 Hz. Hence, these can be used to considerably increase the accuracy of contactless VP measurements [29].

In this publication, a 24GHz continuous-wave radar system for relative distance measurements is presented. After explaining the physiological properties of the target VP, a prototype system is shown as well as tailored algorithms, both to maximize the signal quality and hence increase the reliability of the system. A clinical study with 30 healthy volunteers compares the proposed radar-based VP assessment with gold standard VP detection by impedance cardiography (ICG) and ECG for heart rate and respiration rate, respectively.

## 2. Physiological Fundamentals

For the design of sensor systems, a profound understanding of the underlying physiological principle is necessary. In the case of the radar-based sensing of VP, the action of heart and lungs are of particular interest, especially with respect to the resulting movements and vibrations of the body’s surface. Both, the cardiovascular and the respiratory physiology, will therefore be explained in detail in the following.

### 2.1. Cardiovascular Physiology

The main task of the heart is to supply all vital organs with blood. Every day, the heart pumps over 7000 L of blood through the human body. Cardiovascular diseases have been the main cause for death in industrialized countries since the beginning of the 20th century [30,31].

**Figure 1:**
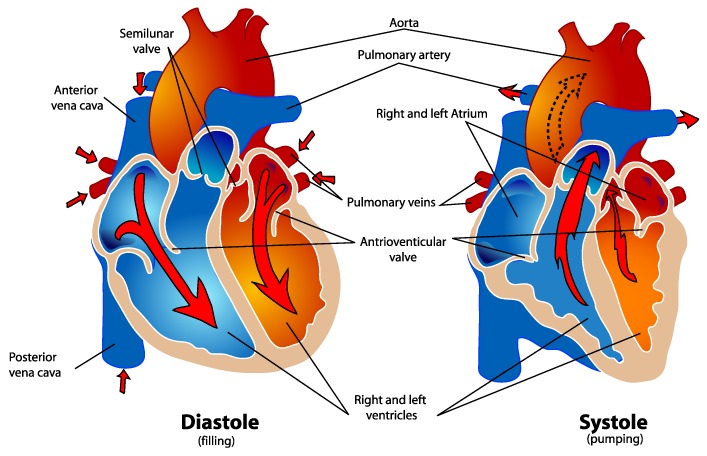
Schematic illustration of the human heart [33].

An illustration of the human heart can be seen in Figure 1. From a functional point of view, the heart consists of two separate pump systems: the left system which pumps the freshly oxygenated blood through the aorta into the systemic circulation of the body and the right system which pumps the blood from the superior and inferior vena cava into the pulmonary circulation through the pulmonary artery. Both systems consist of an atrium and a main chamber which is called ventricle. During each cardiac cycle, both ventricles of the heart contract simultaneously. The pumping effect is based on the rhythmic sequence of contraction (systole) and relaxation (diastole) of the heart. In the diastole, the ventricles are filled with blood. During the systole about 2/3 of the blood volume in the ventricles are ejected. The left half of the heart is usually much more muscular since it has to pump against a much higher pressure in the subsequent circulation [30,32].

During each cardiac cycle sounds caused by the action of the heart muscle can be observed. Physiological heart sounds which occur in a healthy state are called heart sounds whereas pathological sounds are called heart murmurs. In healthy adults, two heart sounds occur. The first heart sound is caused by the contraction of the muscle during the systole and the closing of the valves. Shortly after its occurrence, a second heart sound results from the closure of the aortic and pulmonary valves. By recording heart sounds, heart rate and hemodynamic properties of the heart can be determined. This allows for the diagnosis of potential cardiovascular diseases [30,32].

### 2.2. Respiration Physiology

When talking about respiration, a distinction between internal and external respiration has to be made. Internal respiration describes the process of oxygen exchange between cells in the body. Since this should not be the focus of the measurements, it is not further dealt with. External respiration is the gas exchange of oxygen and carbon dioxide at the level of the alveoli in the lungs. For an optimal saturation of oxygen in the blood humans have two lungs with an optimized structure for gas exchange. A full breathing cycle consists of an inspiration and an expiration phase. During inhalation, fresh air is drawn into the lungs through the nose or mouth. Diffusion occurs through the walls of the alveoli, where oxygen is absorbed into the blood and carbon dioxide is transferred from the blood to the inhaled air. Finally, the air is exhaled again through the mouth or nose [34].

At rest, a healthy adult is expected to have a normal respiratory rate of 12–25 breaths per minute (BrPM), whereas values below 12 BrPM are referred to as bradypnea and those above 25 BrPM as tachypnea [35,36].

**Figure 2:**
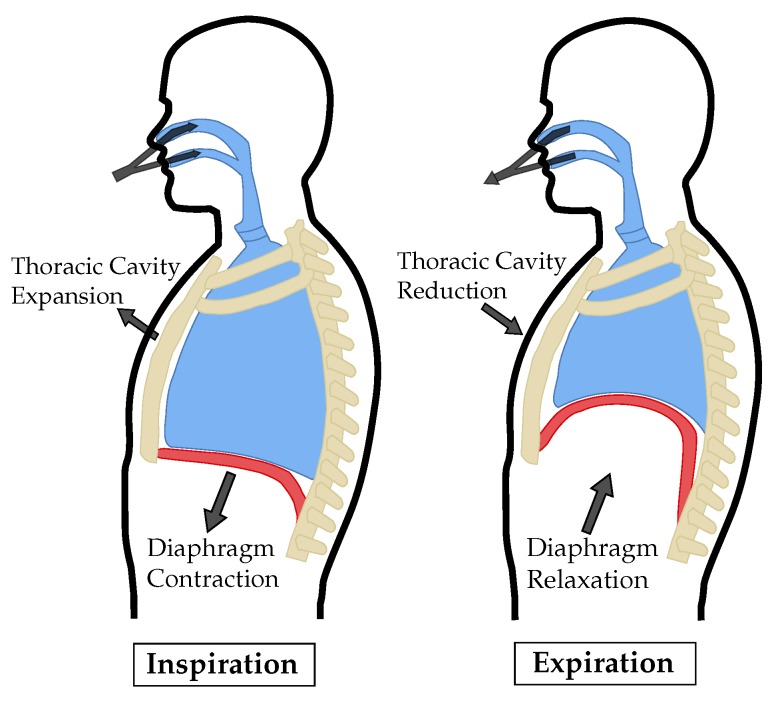
Simplified illustration of the physical motion during inspiration and expiration [32].

While breathing at rest, only inspiration requires muscular activity. Expiration is mainly caused by the elastic recoil of the lungs and relaxing muscles. A simplified illustration of the mechanical action of inspiration and expiration can be seen in Figure 2. The diaphragm and the intercostal muscles contract during inhalation. By raising the chest and tensioning the diaphragm, the thoracic volume increases, resulting in a subatmospheric pressure that allows air to flow through the airways into the lungs. When exhaling msucular activity of the diaphragm and intercostal muscles ceases and the lungs’ volumes are reduced by elastic recoil of their tissue. This causes the air to flow out of the lungs by an increased pressure [37].

## 3. System Concept

The system used for the VP sensing application is a bistatic continuous wave (CW) radar based on the six-port architecture and provides highly precise relative distance measurements. It is tailored to the target application in terms of radio frequency (RF) properties and interfacing. These aspects will be explained in detail in the following, a prototype will be shown and evaluation measurements will be presented.

### 3.1. RF Concept and Design

**Figure 3:**
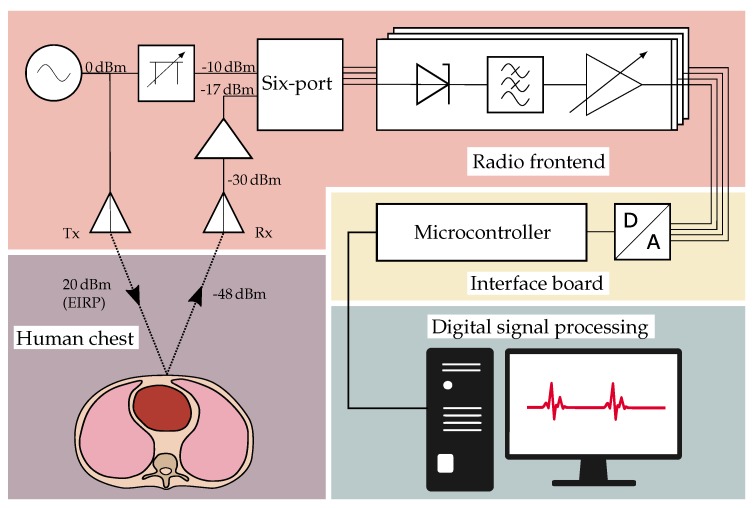
Block diagram of the vital parameter radar sensing system.

The radar system operates at a single frequency of 24GHz. As shown in Figure 3, a voltage-controlled oscillator (VCO) from Infineon Technologies, Neubiberg, Germany (BGT24MTR11) is used to generate the RF signal to be sent via the transmit (Tx) antenna. In order to ensure a low phase noise and a stable output frequency, a phase-locked loop (PLL) from Analog Devices, Norwood, MA, USA (ADF4159) is used in integer mode. The electromagnetic wave transmitted by the system is reflected from the PUT’s chest and then captured by the receive (Rx) antenna. After amplification by a low noise amplifier (LNA) from SiliconRadar, Frankfurt/Oder, Germany (LNA_24_004) with variable gain, the signal enters the six-port receiver structure, where it is superimposed with the reference signal. This is provided by an additional output of the VCO and passes a digitally adjustable attenuator (Analog Devices HMC1018) to control its power level. The four outputs of the six-port structure are connected to active power detectors (Analog Devices ADL6010) and the resulting low frequency (LF) signals are filtered, amplified and digitized by the interface board.

#### 3.1.1. Six-Port Receiver

**Figure 4:**
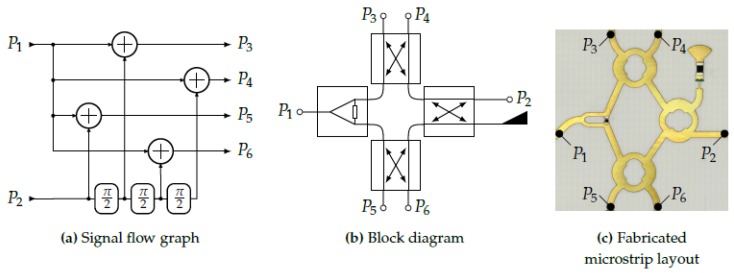
The six-port receiver: Theoretic concept and planar realization.

Being the core component of the radar system, the overall performance strongly depends on the properties of the receiver architecture. In this case, a so-called six-port structure superimposes its two input signals, the reference signal ($P_{1}$) and the receive signal ($P_{2}$), and introduces different phase shifts at the outputs (see Figure 4). For a compact realization, the structure can be implemented by a planar microstrip line layout. Therefore, a Wilkinson power divider and three branchline couplers can be used. Since this structure is purely passive, it does not introduce nonlinearities which might distort the signal. In the next step, the power of the signals at the four output ports, denoted as $P_{3,\cdots ,6}$, is converted to proportional voltages ($B_{3,\cdots ,6}$). This is done by RF power detectors based on nonlinear semiconductor devices, such as Schottky diodes, which convert the power of the RF signal into a proportional DC voltage. For an increased temperature stability and linearity of the transfer curve, commercial active integrated circuits like the chosen Analog Devices ADL6010 contain additional linearizer stages after the power detector.

#### 3.1.2. Antenna Design

Since the architecture of the radar system is bistatic, two separate antennas are required for Tx and Rx, respectively. As indicated in Figure 3, both antennas have to focus on the same spot on the human body to maximize the receive power and hence the signal-to-noise ratio (SNR). Since the antennas are placed next to each other, their beams have to be tilted towards the common focal point. In this case, the required inclination angle of the antenna beam is $\pm 10^{\circ }$ for Tx and Rx antenna, respectively, at a target distance of 40cm. Further requirements on the antennas are a feasibly high gain of about 19dBi and a symmetric radiation diagram with respect to E and H plane. To ease the integration into a compact housing, a planar design is preferred.

The developed antenna prototype is a two dimensional planar patch array on a Rogers RO4350B RF-substrate and consists of 72 radiating patches, which are arranged in nine columns of eight elements each. The size of the elements in each column is tapered with a Taylor window to optimize the radiation pattern. The feed network connecting the columns makes use of T-junctions as power splitters and introduces the required phase shift of $30^{\circ }$ between the columns by curvy delay lines of different amplitudes. The chosen topology with serial delay lines leads to a very compact design, but at the same time sums up the errors of subsequent delays, which requires accurate 3D field simulations and optimizations of the entire structure. Another benefit of the feed network is its power distribution, delivering less power to the array columns the further away they are from the central element. This inherent power tapering helps to reduce the side lobe levels by slightly increasing the main lobe width.

**Figure 5:**
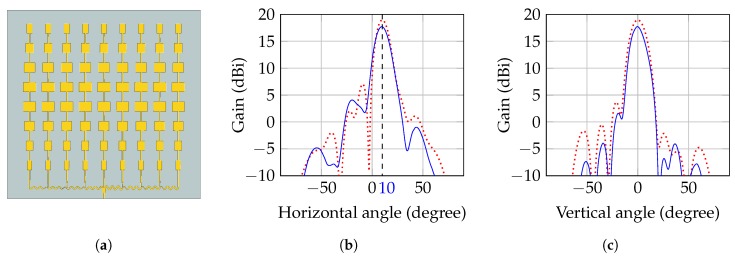
Antenna layout and radiation patterns of the series-fed patch array. (**a**) Antenna layout used for the field simulations; (**b**) Simulated (dashed) and measured (solid) radiation pattern in horizontal plane; (**c**) Simulated (dashed) and measured (solid) radiation pattern in vertical plane at $10^{\circ }$ inclination.

As shown in Figure 5, the fabricated antenna showed the desired tilt of the beam of $10^{\circ }$ and a gain of 17.7dBi with the side lobes being at least 14dB below the main lobe level.

#### 3.1.3. Link Budget

Knowing the characteristic parameters of the antenna, the link budget of the system can be estimated to verify the system concept and to determine the required gain of the receiver. The signal generated by the VCO has an adjustable output power of maximum 11dBm [38], which is attenuated by 3dB due to the losses on the printed circuit board (PCB), connectors and cable. The effective signal strength is then enhanced by the antenna gain (17.7dBi). To make full use of the limit of 20dBm equivalent isotropically radiated power (EIRP) given by the regulations of the industrial, scientific and medical (ISM) band, the VCO output power was adjusted accordingly. The free space attenuation at the given distance of 40cm can be calculated to be 58dB including the return path and assuming an ideally reflective target. From simulations with BioModels from CST Microwave Studio, a value of 10dB was obtained as additional attenuation due to the energy that is absorbed by the human body and the energy radiated in other directions than the receiving antenna, which could be confirmed by measurement. After the LNA with a maximum gain of 15dBm, an Rx power level of $P_{2}=-17,3\;\mathrm{dBm}$ is incident on the input of the six-port receiver. The attenuation of the reference signal $P_{1}$ is adjusted such that the maximum power at ports 3–6 equals about −10dBm, which is well in the linear region of the detector transfer curve.

#### 3.1.4. Analog Baseband

The voltages obtained by the detector, also called baseband voltages $B_{3,\cdots ,6}$, are then further conditioned in the analog domain. Because the power detectors provide a large bandwidth, analog filters are required to reduce the noise, which is outside the frequency band of interest. Therefore, two first-order lowpass filters with a cutoff frequency of 120Hz are added before and after an amplifier, respectively. The amplifier has a digitally adjustable gain and controls the different baseband voltages such that they will make full use of the following analog-to-digital converter’s (ADC) dynamic range of 0–4 V.

### 3.2. Interface Board

In order to further process the raw measurement signals, the RF frontend is connected to an interface board, which contains an ADC (ADS1298 from Texas Instruments, Dallas, TX, USA) with a maximum resolution of 24 bits to digitize the raw data. Using its serial peripheral interface (SPI), the data are handed over to the Infineon XMC4500 microcontroller, arranged into UDP packets and sent to a computer via the Ethernet interface. Alternatively, the data can be directly saved on a memory card. Moreover, the microcontroller is used to address all further components of the RF frontend such as VCO and amplifiers. Two external status lights indicate whether the system is transmitting RF power (green led) or an error has occurred (red led), which facilitates the use of the system as a medical instrumentation device in a clinical environment. To ease the alignment of the system, the interface board controls a class 1 laser, which is placed in the center between Tx and Rx antenna and projects the shape of a cross onto the target, indicating the focus of the antennas. To mark the occurrence of specific events in the data, such as interventions during a proband study, a pushbutton can be connected to the interface board and is digitized simultaneously with the radar signals. The system is powered by an AC/DC-converter, which guarantees an isolation of 4kV, meeting the requirements for medical devices as given by the common standards of IEC 60601-1-2. To avoid distortions caused by the grid, multiple stages of low-dropout regulators (LDOs) filter the supply and suppress unwanted noise.

### 3.3. Fabricated Prototype

**Figure 6:**
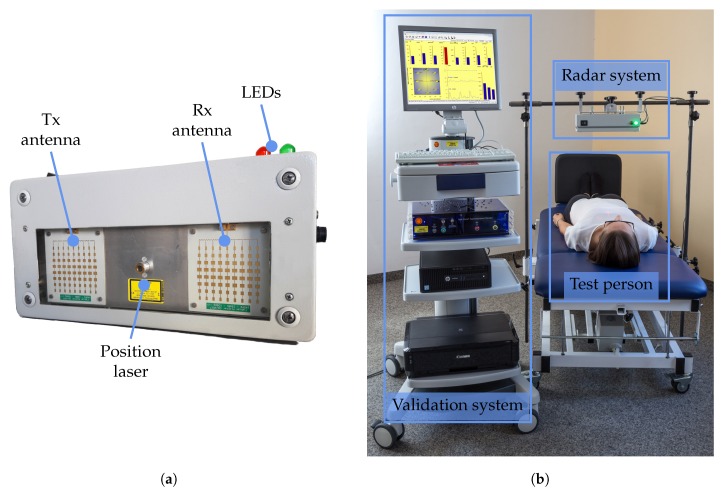
Photographs of prototype and validation systems. (**a**) Photograph of the radar system prototyp; (**b**) Photograph of the study setup.

The final prototype of the system can be seen in Figure 6a. The entire system is integrated into a metal housing of $15\times 8\times 30\;{\mathrm{cm}}^{3}$ with a window of acrylic glass for antennas and laser. On the one hand, this ensures an electromagnetic shielding from interfering radio devices. On the other, it increases its robustness and protects the system from aggressive solvents used for disinfection in the clinical environment.

## 4. Signal Processing

**Figure 7:**
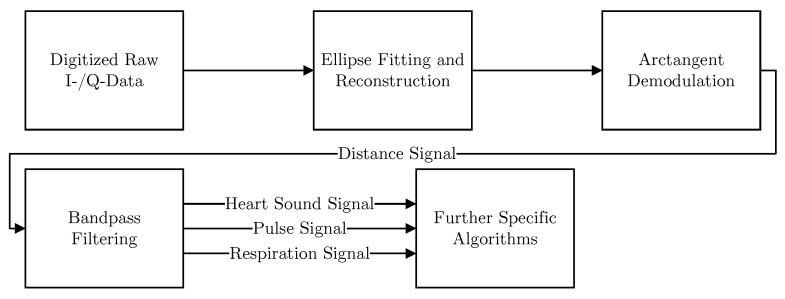
A flowchart showing the overall signal processing steps.

An overall signal processing flowchart is shown in Figure 7. At the beginning, the raw signals are digitized and an ellipse fitting algorithm is used to compensate for non-idealities. Afterwards, arctangent demodulation is employed to retrieve the relative distance signal from the phase information. This distance signal is then filtered using different bandpass filters to retrieve the according components of the radar signal. Specific algorithms are finally utilized for further analysis. All steps will be explained in detail in the following.

### 4.1. Distance Signal Reconstruction

According to theory [39], the time-dependent relative target displacement d(t) is linearly related to the change in phase φ(t) between sent and received RF signal of known wavelength λ:

(1) $$d\left(t\right)=\frac{\lambda }{2}\;\cdot \;\frac{\varphi (t)}{2\pi }.$$

The required measurement of the phase is done by the six-port receiver and the subsequent detectors. It can be shown that the output signals form the real and imaginary part of a complex signal $\underline{Z}\left(t\right)$, which corresponds to the complex baseband of a quadrature down-conversion:

(2) $$\underline{Z}\left(t\right)=\left[B_{5}\left(t\right)-B_{6}\left(t\right)\right]+j\left[B_{3}\left(t\right)-B_{4}\left(t\right)\right].$$

Ideally, the argument of this number equals the desired phase angle:

(3) $$\varphi \left(t\right)=\arg\left\{\underline{Z}\left(t\right)\right\}=\arctan\left(\frac{B_{3}\left(t\right)-B_{4}\left(t\right)}{B_{5}\left(t\right)-B_{6}\left(t\right)}\right).$$

If the radar target moves radially with a constant velocity, the phase will change linearly and hence $\underline{Z}\left(t\right)$ represents a circle centered around the origin. However, cross-talk between transmitting and receiving antennas and offset errors will move the circle center. Moreover, gain errors will change the shape of the circle to an ellipse. In order to reconstruct the true phase from the measured raw data, an ellipse is fitted on measured samples in I/Q domain. Using the extracted parameters of the fitted ellipse, the data can be normalized to a unit circle around the coordinate origin [40], of which the phase angle can then be translated into a displacement.

### 4.2. Respiration Signal Analysis

After the radar signal has been normalized, the resulting phase can be converted into a relative distance change. This signal then reflects the distance change between radar and PUT. Considering a PUT at rest, the distance signal consists of a respiration and heartbeat motion. First, the slow changing respiration with distance changes of 4–12 mm is analyzed. It is necessary to distinguish whether apnea phases are to be detected in the signal or whether the respiratory rate of the PUT is to be determined. In a lowpass filtered distance signal the apnea phases can be recognized easily, whereas in a bandpass filtered signal the respiratory rate can be determined more reliably.

**Figure 8:**
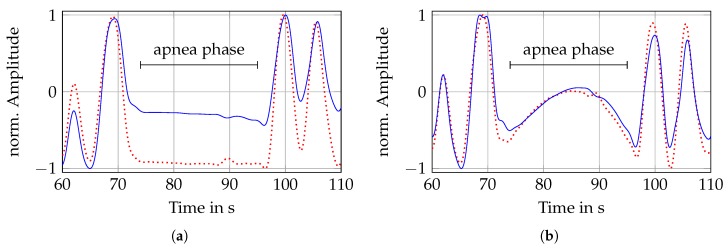
Comparison of low- and bandpass filtered respiration signals. (**b**) Reference (dashed) and radar (solid) respiration signal after applying a lowpass filter; (**b**) Reference (dashed) and radar (solid) respiration signal after applying a bandpass filter.

Taking the respiratory rate of a healthy person of 12–20 BrPM into account, the cutoff frequencies of the bandpass filter are selected at 0.05 Hz and 0.5 Hz for a corresponding range of 3–30 BrPM. Accordingly, for the lowpass filter only the upper cutoff frequency of 0.5 Hz, corresponding to 30 BrPM, is used. Both filters are designed with a fourth order Butterworth characteristic. An exemplary section of apnea is shown in Figure 8, here only the frequency response differs.

Figure 8a,b show the filtered reference ICG and radar distance signals. The reference system (Task Force Monitor) uses two band electrodes placed at the neck and the upper abdomen, respectively. These are used to insert an alternating current at a frequency of 40 kHz and to measure the resulting voltage across the thorax. The ratio of both quantities yields the time-dependent transthoracic impedance, from which the respiratory signal can be extracted by appropriate filtering [41].

Whereas in Figure 8a a fourth order Butterworth lowpass filter is used, Figure 8b shows the result of using a fourth order Butterworth bandpass filter. If one compares the lowpass filtered breathing of the two sensors in Figure 8a, one can see that the apnea area is visible in both signals. The only difference between both filters is the intensity of the inspiration, because an error in unwrapping the phase occurred in the radar signal, which led to a constant offset error. The apnea phase in the bandpass filtered signals in Figure 8b can no longer be identified that easily, because constant components are filtered out. In addition, the offset error is not significant anymore.

After filtering, the breath signals are split into 30 s windows and are further analyzed. In order to compare different methods for respiratory rate calculation, a measurement is divided into windows and for each of these windows the respiratory rate is determined with the four listed methods:

- **Autocorrelation**: With the help of the autocorrelation (ACF) periodicity can be found in the respiration signal. After calculating the ACF, ${\hat{R}}_{xx}\left(m\right)$ corresponds to:
  (4) $${\hat{R}}_{xx}\left(m\right)=\left\{\begin{array}{cc} \;\sum_{n=0}^{N-m-1}x_{n+m}\;\cdot \;x_{n}^{*}, & m\geq 0,\\ {\hat{R}}_{xx}^{*}\left(-m\right), & m<0, \end{array}\right.$$
  where *x* is the windowed breathing signal with length *N*. Subsequently, the respiration rate can be determined by selecting the largest peak in the range of possible respiration durations. In case of the filter limits selected here, the range spans from 3 s to 30 s, which is converted into points using the known sampling frequency. The extracted lag is then converted into the respiratory rate ${\mathrm{RR}}_{ACF}$.
- **Peaksearch**: Using the Matlab internal function ’findpeaks’ with specifying a minimum peak distance and prominence all minima and maxima can be found. The minimum distance of two peaks has to include at least the highest respiration frequency. Therefore, a minimum distance of 3 s is chosen. By differentiation of the minima and maxima locations the durations for both extrema are calculated. Finally, the values are averaged and translated to the respiration rate ${\mathrm{RR}}_{PS}$.
- **Zero crossing**: Considering the bandpass filtered signal, the respiration is centered around zero. Therefore, the zero crossings (ZCs) can be determined by (5) in order to calculate the respiration rate:
  (5) $$\mathrm{ZC}=\sum_{n=1}^{N-1}1_{A}\left(\left(x_{n}\right)\left(x_{n+1}\right)<0\right).$$
  Saving all locations of ZC occurings ${\mathrm{ZC}}_{loc}$ in the given window, the respiration rate ${\mathrm{RR}}_{ZC}$ can be determined by converting twice the mean value of the differentiated locations into BrPM:
  (6) $${\mathrm{RR}}_{ZC}=\frac{60\;\cdot \;Fs}{2\bar{\Delta {\mathrm{ZC}}_{loc}}}.$$
- **Fast Fourier transform**: The frequency components of a time signal can additionally be determined using the fast Fourier transform (FFT). Moreover, all signals are windowed with a Hann window of the same length before applying the FFT. The frequency spectrum is calculated for each window and the maximum of the spectrum in the range from 0.05 Hz to 0.5 Hz is determined. Afterwards it is converted to ${\mathrm{RR}}_{FFT}$.

After determining the respiration rates, the RMSE is calculated for each measurement using:

(7) $$\mathrm{RMSE}=\sqrt{\frac{\sum_{n=1}^{N}{\left({\hat{x}}_{n}-x_{n}\right)}^{2}}{N}},$$

with $\hat{x}$ as the respiration rate of the *N* reference breathing windows and *x* the rate of the *N* radar breathing windows. Next to the respiratory rate the cross-correlation coefficient of radar and reference breathing is calculated as a measure for similarity. Despite synchronization, distance and impedance are different physical parameters that might have an offset between each other. The correlation is calculated according to (4), whereby two different signals are correlated, not one signal with itself. In the end the maximum value of the correlation ${\hat{R}}_{xy,max}$ is determined and the corresponding lag $m_{max}$.

### 4.3. Heartbeat Signal Analysis

Recently, it has been shown that radar systems are able to perform contactless measurement of heart sounds by choosing the right cutoff frequencies [29]. This can be achieved by filtering the raw distance signal between 16 Hz and 80 Hz. Furthermore, Will et al. stated that the analysis of heart sounds results in higher accuracies compared to pulse waves or rather sphygmograms [42] when analyzing the heart rate. Therefore, this paper will compare the performance of both methods when compared to a reference ECG. As a first step, the so called interbeat intervalls (IBIs) are calculated using both the sphygmograms and the heart sounds. To do so, two state-of-the-art algorithms are used: the hidden semi-Markov model (HSMM) segmentation algorithm of [43] in case of heart sounds and the template matching algorithm of [44] in case of sphygmograms. Both algorithms predict points in time where single heartbeats are detected. The IBIs constitute the distances between successive heartbeats which in case of the ECG are calculated as the distance between the R-peaks. By comparing the predicted and reference IBI values *I* and $I_{ECG}$, the RMSE can be determined:

(8) $$\mathrm{RMSE}=\sqrt{\frac{\sum_{n=1}^{N}{\left(I_{n,ECG}-I_{n}\right)}^{2}}{N}}.$$

The smaller the RMSE value, the closer the IBI values are to the reference which in turn indicates a high accuracy. If confirmed that the segmentation of the heart sounds performs better than the analysis of the sphygmograms, additional scores are calculated. Therefore, a threshold needs to be determined for which heartbeats are labeled as detected correctly. In case a single heart sound is identified in a range of 75 ms around a reference ECG R-peak, it is marked as true positive (TP). This value is chosen well within the limits of inaccuracy permitted in certified medical products (150 ms [23]). The even narrower margin of half the tolerance reflects the accuracy of radar-based heart sound detection. If no heart sound is detected in this area, a false negative (FN) is counted. If no reference peak is around a predicted heart sound, it is labeled as false positive (FP). Using the sum of these values, precision and recall can be calculated:

(9) $$\begin{array}{c} \mathrm{Precision}=\frac{\mathrm{TP}}{\mathrm{TP}+\mathrm{FP}}, \end{array}$$

(10) $$\begin{array}{c} \mathrm{Recall}=\frac{\mathrm{TP}}{\mathrm{TP}+\mathrm{FN}}. \end{array}$$

Using those values, the F1 score is determined which constitutes the harmonic mean of these both parameters:

(11) $$F1=2\;\cdot \;\frac{\mathrm{Precision}\;\cdot \;\mathrm{Recall}}{\mathrm{Precision}+\mathrm{Recall}}.$$

**Figure 9:**
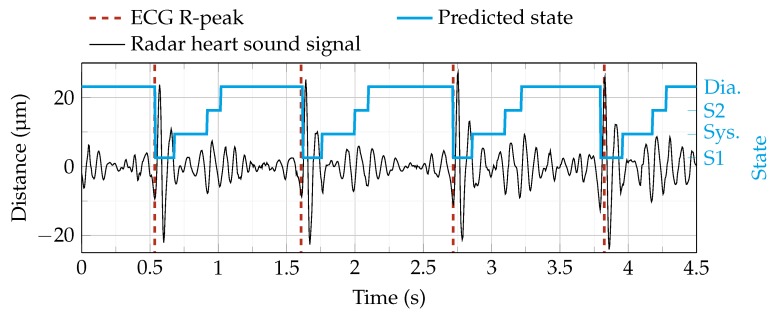
Segmentation of a radar heart sound signal using the hidden semi-Markov model (HSMM) algorithm [43]. The segmentation procedure predicts four states (blue line): First heart sound (S1), systole (Sys.), second heart sound (S2) and diastole (Dia.). The ECG R-peaks are plotted as a visual reference.

Figure 9 shows an example of heart sound segmentation using the HSMM algorithm. The algorithm predicts four phases: S1, systole, S2 and diastole. If the start of S1 is detected in the tolerance range around the R-peak, a TP is counted.

## 5. Validation Study

For the clinical validation, the radar system was mounted on an electrically tiltable table from CNSystems by a custom-made fixture. This allows an adjustment of the radar system’s position to adapt to the physical properties of different test persons such as body size. The entire setup, including the mounted radar system, can be seen in Figure 6b.

As reference, the radar system was synchronized with a medically approved gold standard device, the Task Force Monitor from CNSystems, Graz, Austria. For this clinical study, its three channel ECG (accuracy: ±5µV) and the ICG electrodes were used to continuously measure heart rate and respiration rate. For the synchronization of both devices, the radar system generates a pseudo random noise sequence, which is sampled simultaneously by an analog input of the Task Force Monitor and the radar system’s ADC. For the retrospective synchronization, both digitized signals are resampled and aligned by shifting the maximum of the cross-correlation of both recorded synchronization sequences to a zero time lag.

To compare the performance of the proposed system to the gold standards, a medical study with 30 healthy participants, 14 male and 16 female, was conducted according to the underlying ethics approval. The age of the randomly chosen subjects was in the range of 21–61 years and their body mass index was between 18.6 to $31.4\;\mathrm{kg}/m^{2}$. All participants gave their informed consent. In a first step, medical staff checked the health state of the participant by means of a questionnaire. Auscultation of the heart was performed prior to measurement to exclude participants with heart valve diseases. Next, the participants laid down on the examination table. Heart beats were recorded by electrocardiography and respiration was monitored by impedance cardiography (Task Force Monitor). The optimal spot for radar measurements was determined by auscultation of the heart and the radar system was aligned accordingly. The measurement itself took 90 min in total but for the sake of consistency, only the data from phases with the subject in the supine position at rest is analyzed in this article. During the entire study, at least one medically trained person and one assistant were present. Data were collected in a pseudonymized manner and processed jointly by engineers and medical staff.

## 6. Results

Having described system, data acquisition and processing, the measurement results will be shown in the following section, starting with the system’s key figures of merit, the achievable precision and accuracy. Then a quantification of the precision of the radar-based VP detection will be presented based on the assessment of all 30 study participants.

### 6.1. Displacement Measurements

**Figure 10:**
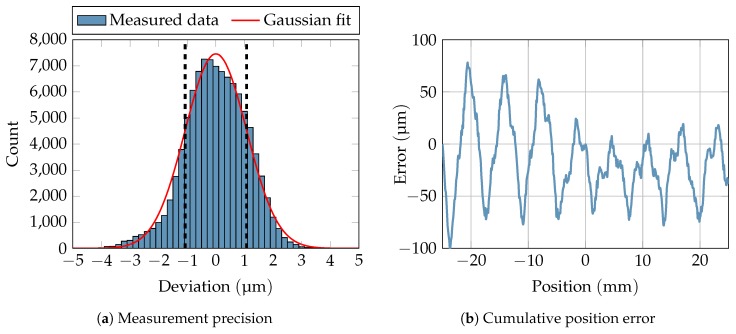
Measurement of precision and cumulative distance error of the presented radar system.

As a measure of the system performance, the achievable precision was measured. A linear stage with a movable metal plate was placed at a distance of 40 cm in front of the radar system. In order to mimic the reflection properties of the human body in the vital parameter sensing application, RF absorbers were stuck to the metal plate to reduce its radar cross section. After powering up the system until it was thermally stable, a linear movement of the stage which serves as reference for the ellipse reconstruction was performed. Subsequently, the stage was stopped and 100,000 samples were recorded, corresponding to a measure time of 50 s. As can be seen in Figure 10a, the samples approximately follow a Gaussian distribution with a standard deviation of 1.07 µm. Drifts of the mean value could not be observed during the measurement.

In order to quantify the cumulative error of a target displacement around a reference point, the linear stage was moved in steps of 100µm. As can be seen in Figure 10b, small absolute errors of less than 100µm occur, if the target is moved by less than ±2.5cm around the reference point. The oscillating errors are caused by linearity errors of the power detectors. The variation of their magnitude is caused by changing operating points of the detectors due to a varying receive power level when the distance between target and system changes. This, however, can be compensated in software by updating the parameters of the fitted ellipse whenever a sudden change in receive power and hence in the operating points is detected.

### 6.2. Respiration

The respiration signals of the two sensor systems were correlated for each measurement according to (4), the results are shown in Table 1. On average, the signals show a correlation of 0.91, which indicates a very high similarity. The lag of the maximum correlation of the respective measurement is 14.7 points on average, which corresponds to a delay of 147 ms at a sampling frequency of 100 Sa/s. Considering the mean value and standard deviation of $m_{max}$, the radar signal is shifted to the left in comparison to the reference, which can be explained by the different physical principles of the measurement methods.

If one compares the RMSE values of the different methods to determine respiration rate, one can see that on average the deviation using ZC is smallest with 0.8 BrPM, using peak search the largest deviations occur with 2.1 BrPM.

Since the deviation of the respiratory rate between reference and radar is smallest when ZC is used, Table 1 also shows the mean respiratory rate of the measurement.

First of all, the RMSE values of the heart rate analysis are compared. While the template matching algorithm achieves an average RMSE of 142.62 ms with a standard deviation of 103.13 ms for all 30 test subjects, the analysis of the heart sounds using the HSMM algorithm significantly outperforms the former with an RMSE of 26.07 ms and a standard deviation of 19.94 ms (p<0.001). Significance is tested on all RMSE values by person. Since these populations are non-Gaussian, the two-sided Kolmogorov-Smirnov test is used. Due to this result, the heart sounds are chosen over the sphygmograms for further analyses.

### 6.3. Heart Rate

Table 2 shows the performance scores for the heartbeat analysis. Each row corresponds to a single test subject. As mentioned in Section 4.3, several scores are calculated for each test subject. F1 score, sensitivity and precision are calculated from the number of TP, FP and FN values. Additionally, the reference as well as the predicted number of single heartbeats and the overall measurement time are given. Using the scores of each test subject, the mean performance can be calculated. This can either be done by calculating the mean over all persons (micro mean) or summing up the number of TP, FP and FN first and calculating the scores based on these values (macro mean). For the first case, the standard deviation is also reported. The F1 micro mean for all test subjects is 93.14% with a standard deviation of 10.74%. The F1 macro mean is slightly lower with 92.82%. The macro means for sensitivity and precision are 92.73% and 92.90%.

F1, sensitivity and precision range from approximately 50% up to 100%. In accordance with common practice, scores from test subjects whose F1 scores deviate more than two standard deviations from the mean F1 value are omitted. In this case, scores from test subjects with an F1 score lower than 71.66% are excluded. This is only the case with test subject 14. The scores after exclusion can be seen in Table 3.

The overall scores clearly improve by up to almost two percentage points. The F1 macro mean is improved from 92.82% to 94.72%. The F1 micro mean now is 94.63% instead of 93.14%. Furthermore, the standard deviation is reduced from 10.74% to 7.14%.

## 7. Conclusions

In this article a system for noncontact measurement of human VP was presented and compared to the gold standard in a clinical study with 30 healthy subjects. A suitable radar system based on an interferometric six-port architecture and tailored antennas were designed and optimized. A precision as low as 1µm with respect to relative distance measurements could hence be achieved. This does not only allow for measuring the displacement of the PUT’s chest during respiration and heartbeat, but also enables the system to detect heart sounds. To research the medical validity of the system, it was connected and synchronized with a medical gold standard device and a clinical study was conducted, showing a high correlation of the radar-based VP assessment with medical gold standard methods. For the respiration analysis, different algorithms based on ACF, ZC, PS and FFT were compared, where ZC clearly outperformed the other methods leading to a mean error as low as 0.828 BrPM for all probands. In case of heart rate detection RMSE of the RR-interval can be decreased from 142.6 ms to 26.07 ms, if heart sounds rather than sphygmograms are used.

Comparing this work to other recent works (Table 4), it can be seen that the number of investigated subjects is clearly outstanding. At the same time, the obtained RMSE of the heart rate is by far the lowest among the publications with radar-based approaches. This result is mainly achieved by the novel method of heart sound analysis in contrast to common sphygmograms, which is enabled by the high measurement precision of the presented system. In terms of respiration measurement, only a few publications use appropriate references, such as respiratory belts. At this point it is also worth mentioning that many authors present systems and algorithms, but do not provide sufficient statistic data regarding the achieved performance. The quantitative validation, however, is an important step in the development of medical devices and was therefore addressed in this publication for both respiration and heart rate.

## Figures and Tables

**Table 1:** Results of respiration analysis. Shown are the maximum correlation value and RMSE values of the four respiration rate determination methods. In addition, the respiration rates of the radar and reference signals are given.

ID	${\hat{R}}_{\mathrm{xy},\max}$	$m_{\max}$ ${}^{a}$	${\mathrm{RMSE}}_{\mathrm{ACF}}$ ${}^{b}$	${\mathrm{RMSE}}_{\mathrm{ZC}}$ ${}^{b}$	${\mathrm{RMSE}}_{\mathrm{PS}}$ ${}^{b}$	${\mathrm{RMSE}}_{\mathrm{FFT}}$ ${}^{b}$	${\mathrm{RR}}_{\mathrm{zc},\mathrm{TFM}}$ ${}^{b}$	${\mathrm{RR}}_{\mathrm{zc},\mathrm{RAD}}$ ${}^{b}$
1	0.952	14	0.224	0.921	2.855	0.063	7.92	8.28
2	0.938	–2	0.919	0.511	1.422	0.407	12.25	12.38
3	0.958	40	0.246	0.575	1.130	0.210	4.77	4.62
4	0.975	17	1.274	0.106	1.078	0.161	10.86	10.86
5	0.979	–12	0.038	0.364	1.580	1.389	8.95	8.99
6	0.916	9	3.866	2.591	4.603	4.173	11.54	12.59
7	0.845	21	2.451	1.245	2.973	0.286	6.01	6.48
8	0.953	5	3.061	1.169	4.424	3.389	13.08	13.52
9	0.940	30	2.754	1.075	1.523	0.299	8.83	9.08
10	0.905	7	0.087	0.100	0.601	0.131	11.99	11.99
11	0.986	19	0.048	0.998	1.114	0.107	17.08	17.26
12	0.937	15	0.095	0.472	0.995	0.151	9.75	9.65
13	0.990	-6	1.570	0.043	1.994	0.640	11.02	11.03
14	0.887	12	0.059	0.083	0.058	0.141	13.35	13.38
15	0.965	8	0.983	0.232	0.230	0.123	8.38	8.45
16	0.965	7	0.052	0.160	0.114	0.100	15.27	15.25
17	0.775	31	1.035	1.026	2.497	3.423	13.81	13.50
18	0.837	18	2.774	2.114	1.332	4.000	12.88	12.53
19	0.940	0	0.760	0.853	1.418	0.903	13.25	13.36
20	0.968	14	0.116	0.139	0.111	0.123	10.52	10.48
21	0.979	0	0.113	0.409	8.527	0.045	17.83	17.91
22	0.472	8	5.149	4.087	5.615	6.619	16.79	13.84
23	0.988	12	0.701	0.068	0.068	0.104	8.68	8.67
24	0.884	17	0.127	0.575	0.107	0.195	13.21	13.10
25	0.914	59	0.949	0.487	3.098	0.957	12.52	12.43
26	0.978	10	1.282	0.085	4.323	0.086	12.48	12.46
27	0.983	12	0.107	0.053	1.616	0.100	10.63	10.61
28	0.972	21	0.380	0.874	2.694	0.436	6.32	5.96
29	0.884	46	0.300	1.005	0.738	0.293	10.37	10.54
30	0.768	10	3.163	2.429	4.391	4.172	11.04	11.81
Mean	0.914	14.7	1.156	0.828	2.108	1.108	11.38	11.37
Std. dev.	0.103	14.9	1.352	0.919	1.971	1.722	3.15	3.03

${}^{a}$ In points, positive: Radar shifted to left; negative: Radar shifted to right. ${}^{b}$ In BrPM.

**Table 2:** Final scores for the heartbeat analysis. The micro mean stands for the direct mean overall test subjects, whereas the macro mean is calculated from the sum of all true positive (TP), false positive (FP) and false negative (FN) values.

ID	F1 Score (%)	Sensitivity (%)	Precision (%)	TP	FP	FN	# R-Peaks ECG ${}^{a}$	# Pred. HB ${}^{b}$	Meas. Time (s) ${}^{c}$
1	95.91	95.97	95.84	691	30	29	729	721	607.6
2	98.27	98.13	98.41	682	11	13	721	694	622.4
3	96.86	95.80	97.94	570	12	25	597	583	601.4
4	98.00	98.00	98.00	685	14	14	702	699	603.1
5	78.81	77.81	79.84	491	124	140	643	617	610.1
6	97.24	97.33	97.15	546	16	15	571	563	610.9
7	74.46	74.10	74.83	452	152	158	647	604	634.9
8	99.04	98.95	99.12	565	5	6	588	572	618.6
9	98.73	98.31	99.15	581	5	10	641	586	649.4
10	85.56	85.28	85.84	394	65	68	491	459	639.1
11	96.60	95.73	97.49	583	15	26	660	601	648.9
12	83.79	83.65	83.92	522	100	102	675	622	648.5
13	99.56	99.50	99.62	791	3	4	801	794	725.6
14	50.06	49.81	50.32	395	390	398	798	785	603.5
15	99.14	99.06	99.22	635	5	6	696	641	648.5
16	97.78	97.58	97.98	484	10	12	504	495	610.6
17	84.92	89.06	81.15	521	121	64	589	643	603.3
18	83.41	83.41	83.41	538	107	107	686	645	636.0
19	95.94	95.64	96.24	461	18	21	484	481	603.1
20	99.84	99.67	100.00	610	0	2	653	610	640.2
21	91.01	90.80	91.21	602	58	61	678	662	613.9
22	95.37	95.20	95.54	535	25	27	619	560	659.8
23	99.68	99.51	99.84	614	1	3	634	616	678.9
24	99.93	99.85	100.00	678	0	1	690	678	610.5
25	98.97	98.97	98.97	671	7	7	697	678	616.9
26	99.29	99.29	99.29	704	5	5	746	711	821.0
27	100.00	100.00	100.00	593	0	0	619	593	627.9
28	97.87	97.49	98.25	505	9	13	528	514	611.9
29	100.00	100.00	100.00	598	0	0	615	598	615.9
30	98.33	97.85	98.82	501	6	11	536	507	626.6
Micro mean	93.14%	93.06%	93.25%	573.3	43.8	44.9	641.3	617.7	635.0
Std. dev.	10.74%	10.72%	10.82%	94.1	78.8	79.1	82.3	82.2	44.2
Macro mean	92.82%	92.73%	92.90%						

${}^{a}$ Number of heartbeats according to reference ECG; ${}^{b}$ Number of predicted heartbeats; ${}^{c}$ Measurement time of test subject.

**Table 3:** Mean values for all test subjects after excluding test subject #14.

	F1 Score (%)	Sensitivity (%)	Precision (%)
Micro mean	94.63	94.55	94.73
Std. dev.	7.14	7.07	7.29
Macro mean	94.72	94.65	94.79

**Table 4:** Comparison of this work to the current state-of-the-art.

	Number of Measured Subjects	Measured Time per Subject (min)	RMSE of Heart Rate (ms)	Correlation of Respiration Rate

this work	30	10	26.07	0.914
[29]	11	>14	44.2	–
[45]	1	20	>200	–
[46]	5	10	45	–
[47]	5	20	>113.2	–
[48]	1	5	–	0.958
[49]	1	5	–	0.9198
[50]	10	1.7	–	0.45 ^a^

${}^{a}$${\mathrm{CO}}_{2}$ sensor as reference leads to poor correlation results.
